# The therapeutically actionable long non-coding RNA ‘*T-RECS*’ is essential to cancer cells’ survival in NRAS/MAPK-driven melanoma

**DOI:** 10.21203/rs.3.rs-1297358/v3

**Published:** 2023-12-01

**Authors:** Valentin Feichtenschlager, Linan Chen, Yixuan James Zheng, Wilson Ho, Martina Sanlorenzo, Igor Vujic, Eleanor Fewings, Albert Lee, Christopher Chen, Ciara Callanan, Kevin Lin, Tiange Qu, Dasha Hohlova, Marin Vujic, Yeonjoo Hwang, Kevin Lai, Stephanie Chen, Thuan Nguyen, Denise P Muñoz, Yoshinori Kohwi, Christian Posch, Adil Daud, Klemens Rappersberger, Terumi Kohwi-Shigematsu, Jean-Philippe Coppé, Susana Ortiz-Urda

**Affiliations:** University of California San Francisco; University of California San Francisco; University of California San Francisco; University of California San Francisco; University of California San Francisco; Department of Dermatology and Venerology, Medical Institution Rudolfstiftung, Vienna, Austria; University of California San Francisco; University of California San Francisco; University of California San Francisco; University of California San Francisco; University of California San Francisco; University of California San Francisco; University of California San Francisco; University of California San Francisco; University of California, San Francisco; University of California San Francisco; University of California San Francisco; University of California San Francisco; University of California at San Francisco; University of California at San Francisco; Sigmund Freud Private University; University of California at San Francisco; Department of Dermatology, Clinic Landstrasse Vienna, Academic Teaching Hospital, Medical University Vienna, Vienna, Austria; University of California at San Francisco; University of California San Francisco; University of California San Francisco

**Keywords:** melanoma, MAPK-pathway, NRAS, Antisense Oligonucleotides, ASO, T-RECS, lncRNA

## Abstract

Finding effective therapeutic targets to treat NRAS-mutated melanoma remains a challenge. Long non-coding RNAs (lncRNAs) recently emerged as essential regulators of tumorigenesis. Using a discovery approach combining experimental models and unbiased computational analysis complemented by validation in patient biospecimens, we identified a nuclear-enriched lncRNA (*AC004540.4*) that is upregulated in NRAS/MAPK-dependent melanoma, and that we named *T-RECS*. Considering potential innovative treatment strategies, we designed antisense oligonucleotides (ASOs) to target *T-RECS. T-RECS* ASOs reduced the growth of melanoma cells and induced apoptotic cell death, while having minimal impacton normal primary melanocytes. Mechanistically, treatment with *T-RECS* ASOs downregulated the activity of pro-survival kinases and reduced the protein stability of hnRNPA2/B1, a pro-oncogenic regulator of MAPK signaling. Using patient- and cell line- derived tumor xenograft mouse models, we demonstrated that systemic treatment with *T-RECS* ASOs significantly suppressed the growth of melanoma tumors, with no noticeable toxicity. ASO-mediated *T-RECS* inhibition represents a promising RNA-targeting approach to improve the outcome of MAPK pathway-activated melanoma.

## Introduction

Melanoma is the deadliest form of skin cancer and its incidence is rising.^1^ Most melanoma tumors harbor oncogenic mutations that activate the MAPK signaling pathway, which regulates cancer cell proliferation and survival.^2^ Even though blocking the MAPK pathway with targeted drugs that inhibit BRAF or MEK kinases has been effective in treating melanoma,^2,3^ acquired or up-front therapeutic resistance is commonly observed in patients.^2^ Discovering molecular mechanisms that may be targeted to reinforce the inhibition of the MAPK pathway or to block parallel pathways that cause melanoma tumors to bypass the effects of MAPK-therapy, is a biomedical priority.^2,4^

A significant proportion of the transcriptome in both normal and diseased cells remains untranslated. The majority of these transcripts are longer than 200 nucleotides and belong to the group of long non-coding RNAs (lncRNAs).^5,6^ LncRNAs are RNA sequences that span ≥200 nucleotides.^6^ Although they do not code for proteins, their expression is tissue specific and is often altered in cancer. LncRNAs can play a role in oncogenesis through various mechanisms, for instance regulating the expression of cancer-specific genes, changing the epigenetic landscape via histone interaction, or serving as splicing factors.^6^ Some lncRNAs can also activate or stabilize proteins through direct binding, which can promote malignancy and influence the response or resistance to therapies.^7,8^ Beyond commonly known lncRNAs such as MALAT1, H19, HOTAIR or SAMMSON,^6,9–11^ more lncRNA transcripts keep being identified as important regulators of cancer. Whether and how lncRNAs may be involved in melanoma progression or in NRAS-mutated/MAPK-dependent cancers, remains largely unknown.^12^

Among recent drug development innovations, RNA-targeting therapies are considered to be particularly promising due to the very broad spectrum of actionable targets they give access to, as well as the highly specific and strong inhibitory effect they can exhibit.^13,14^ Multiple RNA-targeting strategies have been shown to induce apoptosis and eliminate tumor cells.^13,14^ A number of RNA-targeting drugs, including Antisense Oligonucleotides (ASOs), are already FDA-approved.^13,14^ Several ASO drug modalities are currently being tested in clinical trials for cancer treatment.^14^

Here, we report on the discovery of a melanoma-associated lncRNA that we named ‘*T-RECS*’, and that is significantly upregulated in NRAS-/BRAF-mutated cell lines and patient tumors compared to normal/non-malignant cells or tissues. *T-RECS*, which stands for ‘Transcript REgulating Cell Survival’, is coded by the gene *AC004540.4* (ENSG00000225792), a lncRNA observed in non-alcoholic fatty liver disease and hepato-carcinoma.^15,16^ Our study shows that reducing the levels of *T-RECS* with ASOs induces apoptosis in cancer cells, restores sensitivity to MAPK-therapy, and suppresses tumor growth in mouse models. Mechanistically, we demonstrate that, unlike other lncRNA inhibiting ASO treatments, *T-RECS*-targeting ASOs specifically and significantly suppress the activity of pro-survival kinases and downregulates the levels of the pro-oncogenic hnRNPA2/B1 protein. Our results establish that the lncRNA *T-RECS* is a novel melanoma vulnerability, and that ASO-based RNA-targeting strategies can be deployed to inhibit such lncRNA dependency. Altogether, ASO-mediated *T-RECS* inhibition represents an unprecedented therapeutic opportunity to treat NRAS-/BRAF-mutated melanoma.

## Results

### MAPK-pathway activated melanoma cells and tumors express high levels of the lncRNA *AC004540.4*

**Figure 1:**
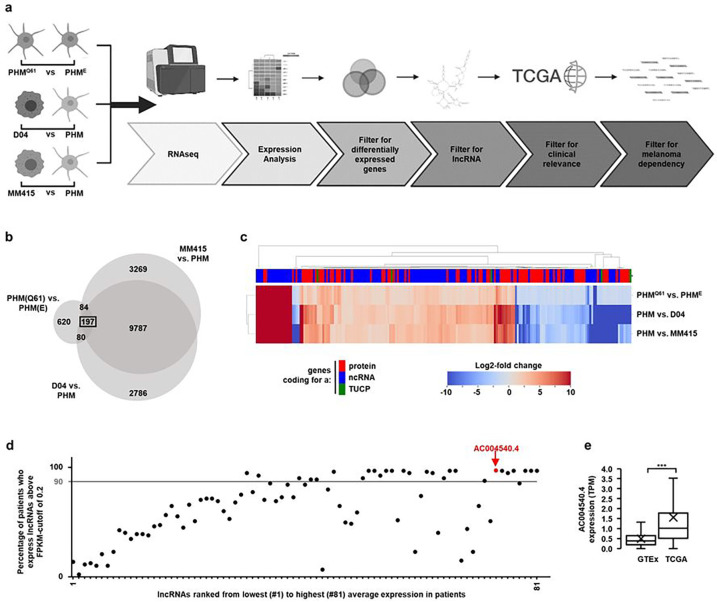
A pipeline to identify lncRNAs that are MAPK-pathway associated, expressed in melanoma patient tissue and essential for melanoma cell-survival. **a)** A schematic illustration of the pipeline steps. RNASeq data of NRAS-mutant primary human melanocytic cells (PHM^Q61^), NRAS-mutant melanoma cell lines (D04, MM415), and wild-type primary human melanocytes (PHM^E^, PHM) were compared to identify lncRNAs whose expression is induced through a MAPK-pathway (NRAS) hyperactivating mutation. **b)** Venn Diagram showing transcriptome intersections the three comparisons PHM^Q61^/PHM^E^, D04/PHM and MM415/PHM. 197 genes were differentially expressed (DE, expression log2-fold change > 1) in all three comparisons. **c**) Expression of the 197 DE genes displayed in a heatmap. Genes were classified in the subgroups Protein-coding genes, non-coding RNA (ncRNA) and Transcripts of unknown coding potential (TUCP). 81 of the 197 DE genes were upregulated lncRNAs **d**) 81 upregulated lncRNAs, ranked from lowest, to highest average expression in patient derived NRAS mutant melanoma from The Cancer Genome Atlas (TCGA, n = 86). 24 lncRNAs were expressed in > 90% (gray bar) of patients. The lncRNA *AC004540.4* was expressed in >97 % of patients and is highlighted in red (Average FPKM = 11.28). **e**) *AC004540.4* expression analysis of non-malignant skin biopsies from the GTEx dataset (n = 1305, mean TPM: 0.49), and BRAF- and NRAS-mutated melanoma tissue from the TCGA-SKCM dataset (n = 366, mean TPM: 1.55) shows that *AC004540.4* is significantly upregulated (*** = p < 0.001) in MAPK-pathway hyperactivated melanoma. The center line represents median expression, the box represents the lower and upper quartiles, the whiskers extend to the furthest value that is less than 1.5 times the interquartile range from the lower and upper quartiles and the mean expression is marked by an ‘X’.

To characterize the expression of lncRNAs in MAPK-driven melanoma, we used D04 and MM415 cell lines, two commonly studied NRAS^Q61^-mutated melanoma models. We also analyzed the transcriptional profile of patient-derived primary melanocytes that were either not transduced or modified to express NRAS^Q61^ or an empty control vector (i.e., primary human melanocytes abbreviated as PHM, PHM^Q61^, PHM^E^). A schematic of the computational process that we used to unbiasedly identify differentially expressed genes including potential lncRNAs, is presented in Fig. 1a. Validation of NRAS^Q61^ expression and downstream activation of the MAPK pathway in PHM^Q61^ cells are provided in **Suppl. Fig. 1a**. We compared the paired-end non-poly A enriched 101-bp RNA-Seq data from PHM^Q61^, D04 and MM415 to the profiles of PHM^E^ or PHM (Fig. 1b; PHM^Q61^ vs. PHM^E^, D04 vs. PHM, M415 vs. PHM; detected genes were classified as coding for a protein or a non-coding RNA or a transcript of unknown coding potential (TUCP)). Out of the >15,000 differently expressed genes we detected, 197 genes were conserved in NRAS^Q61^ expressing PHM^Q61^, D04 and MM415 cells (Fig. 1c, **Suppl. Fig. 1b**). Among these 197 genes, 81 were upregulated lncRNAs.

To hone in on lncRNAs that may be most clinically relevant, we computationally mined the Cancer Genome Atlas - Skin Cutaneous Melanoma (TCGA-SKCM) dataset and found that 24 of these 81 lncRNAs were also expressed in more than 90% of patients’ melanomas harboring an NRAS-mutation (Fig. 1d; FPKM-values > 0.2). In particular, a lncRNA identified as *AC004540.4* (Ensembl Gene ID: ENSG00000225792) was expressed in >97% of patients’ melanomas, with an average FPKM of 11.28 (Fig. 1d; red highlight). *AC004540.4* is located on chromosome 7, expressed as two isoforms, and detectable in some normal tissues (**Suppl. Fig. 2a-b**; Genotype-Tissue Expression (GTEx) database); *AC004540.4* is not conserved in other species. We compared the expression level of *AC004540.4* in biospecimens collected from healthy, non-cancerous skin samples banked in the GTEx database (n=1,305 patient tissues), versus the collection of NRAS- or BRAF-mutated melanoma tumors available in the TCGA database (n=366 patient tissues). We found that the lncRNA *AC004540.4* was significantly more expressed in MAPK pathway-mutated melanomas than in normal skin tissues (Fig. 1e; p < 0.001).

### *AC004540.4*-inhibition reduces melanoma cell growth

**Figure 2:**
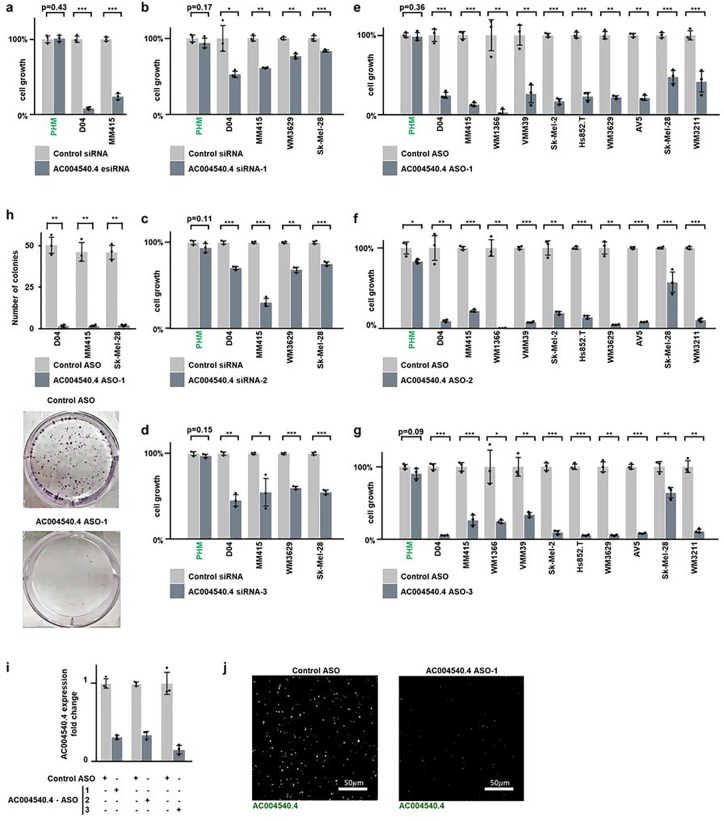
*AC004540.4*-inhibition reduces melanoma cell growth. **a**) *AC004540.4*was targeted using Endoribonuclease prepared siRNA (esiRNA), which caused significant inhibition of cell growth in the melanoma cell lines D04 and MM415. Data were normalized to treatment with non-targeting pooled Control siRNA. **b - d**) *AC004540.4* was targeted using three different siRNA sequences, which caused significant inhibition of cell growth in the melanoma cell lines D04, MM415, WM3629 and Sk-Mel-28. Data were normalized to treatment with non-targeting pooled Control siRNA, average cell growth inhibition with *AC004540.4*siRNA treatment was 39 % and is shown for AC004540.4siRNA-1 in **b**), siRNA-2 in **c**) and siRNA-3 in **d**). **e - g**) Using three different *AC004540.4*-targeting ASO sequences caused significant inhibition of cell growth in the melanoma cell lines D04, MM415, WM1366, VMM39, Sk-Mel-2, WM3629, Sk-Mel-28, WM3211 and the primary derived melanoma cell lines Hs852T and AV5. Data were normalized to treatment with non-targeting Control ASO, average cell growth inhibition with *AC004540.4*ASO-treatment was 80 % and is shown for *AC004540.4* ASO-1 in e), ASO-2 in **f**) and ASO-3 in **g**). *AC004540.4*-targeting oligonucleotide treatment had minimal impact on cell growth of primary human melanocytes (PHM), as shown by far-left bar-pairs in panels **a – g**). **h**) *AC004540.4* ASO treatment inhibited colony formation in melanoma. Top: *AC004540.4*ASO-1 treatment significantly reduced colony formation in the D04 (p = 0.002), MM415 (p = 0.004) and Sk-Mel-28 (p = 0.002) melanoma cell lines compared to treatment with non-targeting Control ASO. Bottom: Representative images of D04 colonies in 6cm dishes after incubation with either *AC004540.4* ASO-1 or Control ASO. i) All three *AC004540.4*ASO constructs specifically and strongly reduced *AC004540.4* RNA levels after 1 day of treatment, when compared to treatment with non-targeting Control ASO in the D04 cell line. j) Representative images of fluorescent signals received from D04 cell pellets treated with either *AC004540.4*ASO-1, or Control ASO shows a strong reduction of fluorescent signal of *AC004540.4*-binding probes. Error bars represent standard deviation and significance is shown as p-values calculated by Student’s t-test. * = p < 0.05, ** = p < 0.01, *** = p < 0.001. Detailed information about cell line characteristics and cell growth reduction can be found in **Suppl. Table 1+2.**

To assess whether the lncRNA *AC004540.4* may confer survival and growth advantage to melanoma cells, we initially used endoribonuclease-prepared siRNA (esiRNA), which is a highly effective method to degrade specific RNA targets.^17^ We found that esiRNA-mediated silencing of *AC004540.4* greatly reduced the growth of D04 and MM415 cell lines (Fig. 2a).

Next, we tested the effects of two RNA-targeting approaches that are clinically relevant: siRNA and antisense oligonucleotides (ASOs), which have received FDA and/or EMA approval for therapy and are commonly used in animal models.^18^ We designed three siRNA sequences and three ASO constructs of the GapmeR type to specifically target *AC004540.4* (respectively *AC004540.4* siRNA-1/-2/-3 and ASO-1/-2/-3 used in Fig. 2b–d and 2e–g; see Methods for details), and tested their effects on cell viability in comparison to non-targeting siRNA or ASO sequences, which served as control for non-specific effects of oligonucleotide treatment (Fig. 2b–g; light vs. dark grey bars). Using a panel of 8 melanoma cell lines (D04, MM415, WM1366, VMM39, Sk-Mel-2, WM3629, Sk-Mel-28, WM3211) and 2 primary melanoma cells (Hs852.T, AV5), we found that these RNA-targeting interventions significantly inhibited cell growth (Fig. 2b–g and **Suppl. Tables 1–2**). While siRNAs targeting *AC004540.4* reduced cell growth by 39% compared to Control siRNA on average (Fig. 2b–d), ASOs had a significantly greater effect, inhibiting cell growth by 80% on average and up to 95% in some instances (Fig. 2e–g). Notably, both siRNAs and ASOs had minimal impact on normal, primary human melanocytes (PHM; far left bar pairs in each graph of Fig. 2b–g), which underlines the specific, anti-melanoma effect of these RNA-targeting interventions while sparing normal melanocytes.

To further determine the efficacy of ASO intervention, we performed clonogenic assays. Compared to Control ASO treatment at equal dose and incubation time, *AC004540.4*-targeting ASO drastically reduced the ability of D04, MM415 and Sk-Mel-28 cells to form colonies (Fig. 2h; p-val < 0.004).

We used quantitative real-time PCR (qRT-PCR) to measure *AC004540.4* levels, and validated that, in comparison to Control ASO, its expression was reduced after *AC004540.4*-targeting ASO treatment in D04 cells (Fig. 2i). Additionally, we used RNA fluorescence in situ hybridization (FISH) staining to visualize ASO-mediated *AC004540.4* knockdown in fixed and paraffin embedded (FFPE) D04 cell pellets (Fig. 2j).

### The lncRNA *AC004540.4* is enriched in the nucleus of melanoma cells

**Figure 3:**
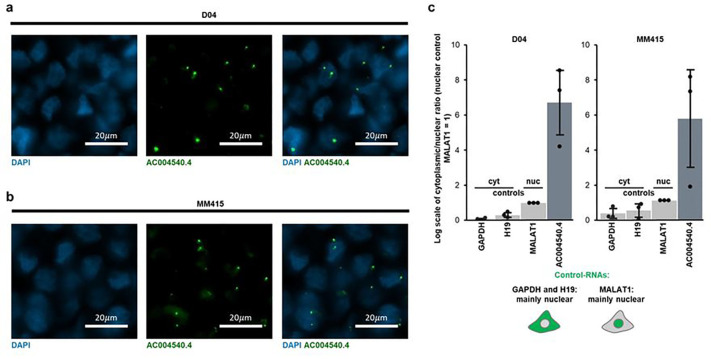
*AC004540.4* is enriched in the nucleus of melanoma cells. **a-b)** Representative images of ISH-signals (RNAscope) derived from D04 (**a**) and MM415 (**b**) cell pellets. Fluorescent signals are either produced by DAPI DNA staining to mark the nuclear regions (blue) or probes that stain the lncRNA *AC004540.4*(green). **c**) Subcellular RNA enrichment analysis of D04 and MM415 cells. QRT-PCR was used to compare the ratio of nuclear versus cytoplasmic RNA levels of *AC004540.4*, GAPDH mRNA and H19 lncRNA, which served as cytoplasmic enriched controls and the MALAT1 lncRNA, which served as nuclear enriched control. The nuclear-to-cytoplasmic ratios of GAPDH, H19 and *AC004540.4* were further normalized to the nuclear-to-cytoplasmic ratio of MALAT1. The data are presented as fold-change of nuclear to cytoplasmic ratio (n=3). Compared to MALAT1, GAPDH (D04: 0.08-fold, MM415: 0.34-fold) and H19 (0.33-fold, MM415: 0.49-fold) present a lower ratio, and *AC004540.4* presents a higher ratio (6.71-fold, MM415: 5.2-fold).

LncRNAs display diverse subcellular distributions, with some primarily located in the nucleus, others in the cytoplasm.^19^ To test whether *AC004540.4* is a predominantly nuclear or cytoplasmic lncRNA, we applied RNA-FISH staining on formalin fixed and paraffin embedded D04 and MM415 cell pellets. Analysis of ISH-stained cell pellets showed that *AC004540.4* is mainly located in the nucleus (Fig. 3a–b). To validate these findings, we performed subcellular fractionation of D04 and MM415 cells followed by RNA extraction and qRT-PCR to compare the ratio of nuclear versus cytoplasmic RNA levels. As control of RNA subcellular location, we used the GAPDH mRNA and H19 lncRNA, which are mainly cytoplasmic^20,21^, and the MALAT1 lncRNA, which is mainly nuclear.^19^ The nuclear-to-cytoplasmic ratios of GAPDH, H19 and *AC004540.4* were further normalized to the nuclear-to-cytoplasmic ratio of MALAT1. We found that *AC004540.4* was mainly enriched in cells’ nucleus, and significantly more nuclear compared to MALAT1 (Fig. 3c; 6.7- and 5.2-fold respectively in D04 and MM415).

### *AC004540.4* promotes cell survival pathways and can be targeted to induce apoptosis

**Figure 4:**
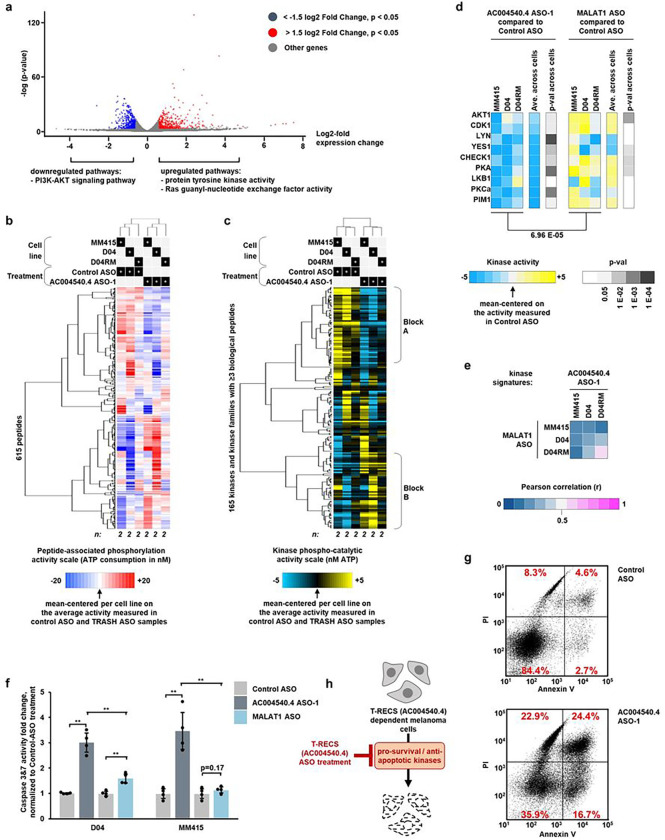
*AC004540.4* promotes cell survival pathways and can be targeted to induce apoptosis. **a**) A volcano plot from RNAseq derived data highlights the differentially expressed (DE) genes from D04 cells treated with either *AC004540.4* ASO, or Control ASO. Cut-off for significance was p < 0.05 and either < −1.5 (downregulated) or > 1.5 (upregulated) log2fold expression level change (n=3). DAVID functional annotation clustering unveiled that "PI3K-AKT signaling pathway" (downregulated), and "protein tyrosine kinase activity" and "Ras guanyl-nucleotide exchange factor activity" (upregulated) were Gene Ontology (GO) terms and pathways found in the top enriched clusters. **b**)Peptide-associated phosphorylation profiles of melanoma cell lines treated with *AC004540.4* ASO, or Control ASO. Unsupervised clustering was applied (using uncentered correlation and average linkage for both peptides/horizontal and samples/vertical). The profile of each sample is the average of two independent assay repeats. **c**) Unsupervised clustering of kinase activity signatures from results shown in previous panel. Kinases for which ≥3 biological peptides are available, are shown. *AC004540.4* inhibition induced a conserved response across cell lines of either activity downregulation (Block A, mainly blue) and upregulation (Block B, mainly yellow). **d**)Kinase activity profiles of a subset of kinases known to promote cell survival by preventing apoptosis is specifically downregulated by *AC004540.4* ASO treatment, as shown by a side-by-side comparison to the effects of inhibiting the distinct pro-oncogenic lncRNA MALAT1 with MALAT1 ASO treatment. **e**) The specificity of the effects of *AC004540.4* ASO treatment on the kinase activity signatures of melanoma cells is assessed in comparison to MALAT1 ASO treatment using Pearson correlation. **f**) As measured by activity levels of the apoptosis markers Caspase-3 & -7, *AC004540.4* ASO treatment induces apoptosis to a significantly stronger extent than MALAT1 ASO treatment in the D04 (3-fold VS 1.6-fold, p < 0.002) and MM415 (3.5-fold VS 1.1-fold, p < 0.006) cell-line, when compared to Control ASO treatment (treatment with 50 nM ASO concentration for 1 day, n=4). Significance is shown as p-values calculated by Student’s t-test. * = p < 0.05, ** = p < 0.01, *** = p < 0.001. Error bars represent the standard deviation. **g**) Dot plot graph of flow cytometric analysis of PI and Annexin V staining after 1 day of ASO-treatment shows increased apoptotic cell death in D04-cells treated with *AC004540.4* ASO compared to Control ASO treatment. Numbers in quadrants (red) show the percentage of vital (bottom left), early apoptotic (bottom right), late apoptotic (top right) and dead (top left) cells relative to the overall population. **h**) Schematic summarizing of the molecular impact of *AC004540.4*(*T-RECS*) ASO-treatment, inducing apoptosis and inhibiting pro-oncogenic kinases in melanoma.

To start uncovering the molecular mechanisms underlying the effect of *AC004540.4*-inhibition and the potential role of *AC004540.4* in MAPK-signaling, we performed gene expression profiling of D04 cells treated with either non-targeting Control ASO or *AC004540.4*-targeting ASO. This comparison revealed that, 72h after treatment, *AC004540.4*-inhibition induced significant changes in 1,067 genes (Fig. 4a; cut off for DE genes: FDR p-values: <0.05 (y-axis); cut off for log2 fold change: > +1.5 or < −1.5 (x-axis)). Further functional annotation and clustering analysis was done using DAVID to search for biologically relevant GO terms and pathways. Terms grouped with “protein tyrosine kinase activity”, “Ras guanyl-nucleotide exchange factor activity”, and “PI3K-AKT signaling pathway” were found among the highest ranked terms (Fig. 4a; see **Suppl. Table 3–5** for details).

In order to more specifically reveal the mechanism of action of ASO-induced *AC004540.4* inhibition, we applied an advanced, high-throughput kinase activity mapping (HT-KAM) assay^22–25^ and measured the changes in kinase activity levels of cells exposed to *AC004540.4*-targeting ASO in comparison to non-targeting Control ASO. Protein extracts from D04, MEK-inhibitor resistant D04 (named D04RM), and MM415 melanoma cell lines treated for 24h with ASOs, were tested on the HT-KAM platform and results were analyzed to establish the functional phospho-fingerprint of samples (see Methods for assay details). The peptide phosphorylation profiles and kinome activity signatures were subjected to unsupervised hierarchical clustering (Fig. 4b–c). We found that *AC004540.4* inhibition induced a conserved response across cell lines (all cells treated with *AC004540.4*-targeting ASO cluster together; Fig. 4c, see top dendrogram, left vs. right). *AC004540.4*-targeting ASO led to conserved kinase activity downregulation (Fig. 4c, Block A, mainly blue) and upregulation (Fig. 4c, Block B, mainly yellow). Remarkably, kinases with pro-survival functions, such as AKT1, CDK1, LYN, YES1, CHEK1, PKA, LKB1, PKCa and PIM1,^26–30^ were significantly less active after ASO-induced *AC004540.4* inhibition in comparison to Control ASO (Fig. 4d, left panel), which directly corroborates the cell growth inhibition effects we observed (Fig. 2a–h).

To demonstrate that these effects are specific to *AC004540.4*-targeting, and not a general effect of inhibiting pro-oncogenic lncRNAs, we treated the same three cell lines with an ASO that targets MALAT1, which is a known pro-oncogenic lncRNA^10,11,19^, and measured changes in kinase activity using HT-KAM. We found that after 24h, the kinome signatures of cells treated with MALAT1 ASO were different from *AC004540.4* ASO treated cells (Fig. 4e; average Pearson correlation *r* < 0.3; all data normalized to non-targeting Control ASO). More specifically, and unlike *AC004540.4* inhibition, we found that targeting MALAT1 did not result in a significant decrease in the activity of pro-survival kinases AKT1, CDK1, LYN, YES1, CHEK1, PKA, LKB1, PKCa and PIM1 (Fig. 4d, right panel). The difference between the activity of these kinases in D04, D04RM and MM415 cells treated with *AC004540.4* ASO versus MALAT1 ASO, was significant (Fig. 4d, left vs. right panel; p-val < 0.00007).

Since these kinases are known to prevent cell death by inhibiting pro-apoptotic pathways, we tested whether targeting *AC004540.4* or MALAT1 with ASOs may differentially induce apoptosis. We found that, after 24h of treatment, *AC004540.4* ASO significantly increased apoptosis in comparison to MALAT1 ASO in D04 and MM415 cells (Fig. 4f; measured by Caspase-3/7 assay; p ≤ 0.002; all data normalized to non-targeting Control ASO). Additionally, we used Annexin V staining to confirm apoptosis induction (Fig. 4g; early apoptotic stage: 2.7% vs. 16.7%, and late apoptotic stage: 4.6% vs. 24.4%, comparing non-targeting Control ASO vs. *AC004540.4*-targeting ASO). Together, our results reveal that in response to *AC004540.4* inhibition, melanoma cells reprogram their kinome in a coordinated fashion, leading to the specific downregulation of cell survival pathways and induction of apoptosis (Fig. 4h; schematic summary). Based on these findings (Fig. 2,4), we named the *AC004540.4* lncRNA “Transcript REgulating Cell Survival” (*T-RECS*).

### *T-RECS* regulates hnRNPA2/B1 protein stability

**Figure 5:**
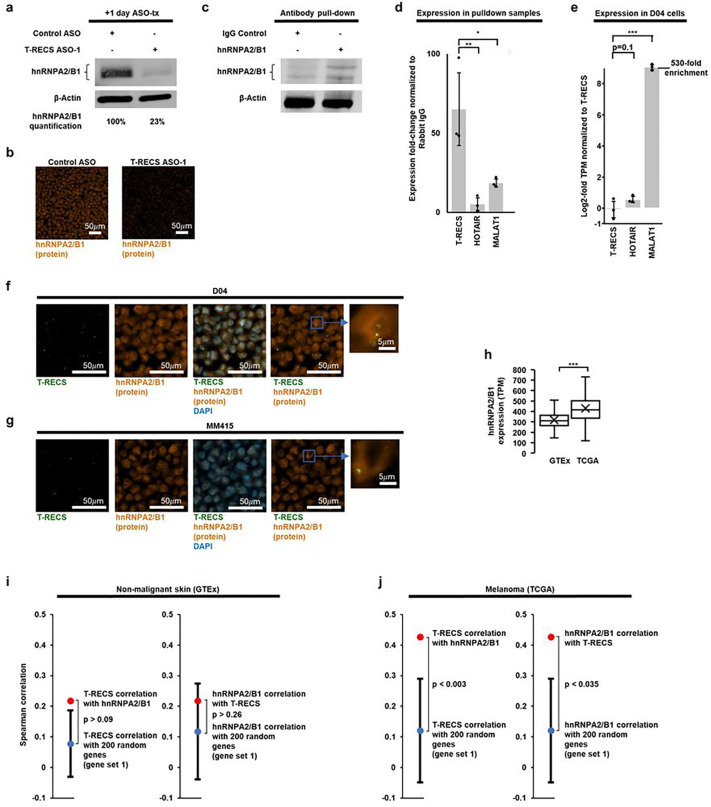
*T-RECS* regulates hnRNPA2/B1 protein stability. **a**) Immunoblotting showing a strong decrease in hnRNPA2/B1 protein levels 1-day after *T-RECS* ASO treatment compared to Control ASO treatment in D04 cell lysate. β-actin served as a loading control. **b**) Representative images of fluorescent signals received from D04 cell pellets treated with either *T-RECS* ASO, or Control ASO shows a strong reduction of fluorescent signal of hnRNPA2/B1-binding antibody. **c**) Immunoblot analysis of D04 cell lysate derived from hnRNPA2/B1 and Rabbit IgG pulldown samples confirms successful hnRNPA2/B1 pulldown. **d**) To account for potential unspecific binding to the RNA-binding protein hnRNPA2/B1, enrichment analysis (calculated at 10%-input) was executed by comparing *T-RECS* enrichment in the hnRNPA2/B1 pulldown samples to other lncRNAs. *T-RECS* was > 65-fold enriched, while HOTAIR (5.4-fold, p = 0.03) and MALAT1 (18-fold, p = 0.049) were significantly less enriched. **e**) Differential expression analysis of RNASeq data unveils that compared to T-RECSexpression, the lncRNA HOTAIR is not significantly higher expressed (1.47-fold increase, p = 0.1) and lncRNA MALAT1 is significantly higher expressed (530-fold increase, p = 0.003) in the D04 cell line. **f**) Representative ISH-derived (RNAscope) fluorescent signals from D04 and **g**) MM415 cell pellets are in green (T-RECS), orange (hnRNPA2/B1 protein), or blue (DAPI-nuclear control). Nuclear compartments with high hnRNPA2/B1 expression levels tend to overlap with areas of higher *T-RECS* expression. **h**) hnRNPA2/B1 expression analysis of non-malignant skin biopsies from the GTEx dataset (n = 1305, mean TPM: 317), and BRAF- and NRAS-mutated melanoma tissue from the TCGA-SKCM dataset (n = 366, mean TPM: 430) shows that hnRNPA2/B1 is significantly upregulated (p < 0.001) in MAPK-pathway hyperactivated melanoma. The center line represents median expression, the box represents the lower and upper quartiles, the whiskers extend to the furthest value that is less than 1.5 times the interquartile range from the lower and upper quartiles and the mean expression is marked by an 'X'. **i**) In healthy-skin patient samples (GTEx, n=1305), the expression correlation coefficient of *T-RECS* and HnRNPA2/B1 (ρ = 0.22, red dot) is not statistically different from the coefficient of *T-RECS* (mean ρ = 0.08, p > 0.09, blue dot in left panel) or HnRNPA2/B1 (mean ρ = 0.12, blue dot in right panel, p > 0.26) compared to a set of 200 randomly chosen genes (gene set 1). **j**) In MAPK-pathway driven melanomas (TCGA, n=366), the expression correlation coefficient of *T-RECS* and HnRNPA2/B1 (ρ = 0.43, red dot) is significantly higher than the coefficient of *T-RECS* (mean ρ = 0.1, blue dot in left panel, p < 0.003) or HnRNPA2/B1 (mean ρ = 0.12, blue dot in right panel p < 0.035) compared to a set of 200 randomly chosen genes (gene set 1). These findings are consistent with those obtained from nine additional gene sets as shown in **Suppl. Fig. 3c-d**. Significance for panel d-e) and h) is shown as p-values calculated by Student’s t-test. * = p < 0.05, ** = p < 0.01, *** = p < 0.001. Significance for panel i-j) was evaluated by comparing the correlation between *T-RECS* and hnRNPA2/B1 with correlations between either *T-RECS* or hnRNPA2/B1 with 200 randomly selected genes and calculating a Z-score. Error bars represent standard deviation.

Considering that lncRNAs can regulate protein-coding genes located nearby their genomic location^31^, we tested whether *T-RECS* may be involved in the expression of HnRNPA2/B1, an oncogene associated with many cancer types including melanoma.^32,33^ We found that *T-RECS* ASO treatment induced a sharp decrease in HnRNPA2/B1 protein levels in D04 cells, as shown by immunoblot and immuno-fluorescence (Fig. 5a,b; up to 75% reduction in HnRNPA2/B1 protein). Since the protein half-life of the HnRNPA2/B1 protein is known to range from several days to up-to-4 weeks in primary human cells^34^, and since we found that directly targeting hnRNPA2/B1 with an ASO induced >50% reduction in RNA level but was accompanied with <20% reduction in protein level of HnRNPA2/B1 after 1 day (**Suppl. Fig. 3a-b**), our results suggest that *T-RECS* may stabilize HnRNPA2/B1 protein through physical binding. To test this, we pulled down HnRNPA2/B1 protein and measured the levels of RNA bound to it. To account for potential unspecific interactions with the RNA-binding protein HnRNPA2/B1^33^, we compared *T-RECS* RNA levels to two other lncRNA transcripts, MALAT1 and HOTAIR^11^. D04 melanoma cell lysates were immuno-precipitated using either IgG control or HnRNPA2/B1 antibodies (Fig. 5c). We found *T-RECS* to be strongly and specifically enriched in the HnRNPA2/B1 pull down lysate (Fig. 5d; left bar). Conversely, HOTAIR and MALAT1 were significantly less co-precipitated with HnRNPA2/B1 (Fig. 5d; two right bars), even though their baseline expression levels were either similar to *T-RECS* (HOTAIR; 1.5-fold), or far more elevated than *T-RECS* (MALAT1; > 500-fold) (Fig. 5e). Next, we used RNAscope combined with immuno-fluorescence to determine whether *T-RECS* and HnRNPA2/B1 colocalized. We found that *T-RECS* and HnRNPA2/B1 signals overlapped in the nuclei of D04 and MM415 cells (Fig. 5f–g). Together, our results indicate that the lncRNA *T-RECS* may directly interact with and stabilize the HnRNPA2/B1 protein (Fig. 5a–g).

### *T-RECS* and hnRNPA2/B1 are co-expressed in melanoma tumors

Since HnRNPA2/B1 can promote tumorigenesis and modulate MAPK-pathway signaling^33^, we asked whether HnRNPA2/B1 may also be upregulated in MAPK pathway-mutated melanoma tumors, and whether it may be correlated with *T-RECS* expression. Comparing expression levels of HnRNPA2/B1 in non-cancerous, patient-derived skin samples from the GTEx database (n=1,305 patient tissues) versus in NRAS- or BRAF-mutated melanoma tumors from the TCGA database (n=366 patient tissues), we found that HnRNPA2/B1 is significantly more expressed in MAPK-driven melanoma (Fig. 5h; p < 0.001). Given that both *T-RECS* and HnRNPA2/B1 genes are significantly upregulated in melanoma, we tested whether their expression is correlated in the GTEx and TCGA datasets. We calculated expression correlation between *T-RECS* and hnRNPA2/B1 and compared it to the correlation of each of the two genes to 10 sets of 200 randomly chosen genes (see Methods for computational details). We found that in non-malignant skin samples (GTEx), there was no significant difference in the correlation of *T-RECS* and hnRNPA2/B1 compared to the correlation of each of them to any 200 random gene set (Fig. 5i; left panels; p > 0.05; only one of the ten different 200-gene sets is shown; all sets are provided in **Suppl. Fig. 3c-d**). However, in melanoma (TCGA), the correlation between *T-RECS* and hnRNPA2/B1 was systematically and significantly higher when compared to the random gene sets (Fig. 5j; right panels). Ranking of expression correlations showed that the correlation of *T-RECS* with hnRNPA2/B1 is significantly higher ranked in TCGA than GTEx when compared to the ten different 200-gene sets (**Suppl. Fig. 3e-f**). These results show that *T-RECS* and hnRNPA2/B1 are not co-expressed in healthy skin, but they are jointly and non-randomly upregulated in melanoma.

### *T-RECS* ASO treatment synergizes with MEKi to inhibit the growth of melanoma cells

**Figure 6:**
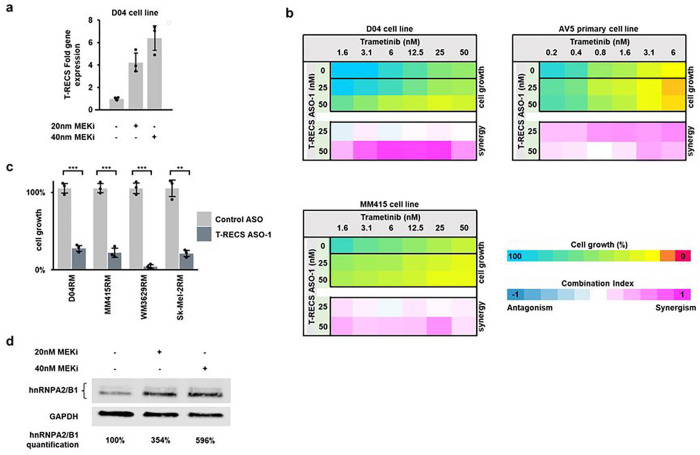
*T-RECS* ASO treatment synergizes with MEKi to inhibit the growth of melanoma cells. **a**) QRT-PCR analysis shows elevated *T-RECS* RNA levels in D04 cells after three days of drug-induced Inhibition of MAPK signaling using the MEKi trametinib (20nM or 40nM), when compared to control, treated with DMSO (n = 3). **b**) D04, MM415 and primary-derived AV5 cells were treated with combinations of *T-RECS* ASO and trametinib. Combination indices (CI) for dual-treatment effects on cell growth were calculated following the Bliss-model (see methods). Synergistic effects were observed in a range of different concentration combinations in all three cell lines. (n = 2) **c**) *T-RECS* ASO treatment significantly inhibited cell growth in the MEKi treatment resistant melanoma cell lines D04RM (p = 0.0002), MM415RM (p = 0.0001), WM3629RM (p = 0.0005) and Sk-Mel-2RM (p = 0.0019). Data were normalized to treatment with non-targeting Control ASO. Significance is shown as p-values calculated by Student’s t-test. * = p < 0.05, ** = p < 0.01, *** = p < 0.001.

Having demonstrated that MAPK-driven melanoma tumors and cell lines depend on the expression of *T-RECS* to grow, we hypothesized that melanoma cells may respond to MAPK-inhibition and overcome therapeutic stress by upregulating *T-RECS* to survive. To test this, we first measured *T-RECS* levels in D04 cell treated for 72 hours with a MEK-inhibitor (MEKi; trametinib) at two different concentrations using qRT-PCR. We found that *T-RECS* was upregulated in MEKi-treated cells compared to control (Fig. 6a; 4-to-6 fold). These results suggest that, as a therapeutic strategy, inhibiting *T-RECS* may augment the response of melanoma cells to MEKi. Using a cell survival assay, we found that combining trametinib with *T-RECS* ASO inhibited the growth of melanoma cell lines and primary melanoma cells more than trametinib alone (i.e., D04, MM415 and AV5), and that this combination was synergistic (Fig. 6b). Furthermore, we established that melanoma cell lines that we induced to become MEKi-resistant (i.e., D04RM, MM415RM, WM3629RM, and Sk-Mel-2RM cells chronically exposed to increasing MEKi doses; see Methods), remained sensitive to *T-RECS* ASO treatment (Fig. 6c). Finally, we found that upregulation of *T-RECS* upon MEKi was accompanied by the stabilization and increased protein levels of hnRNPA2/B1 (Fig. 6d; 3.5-to-6 fold). Together, our results indicate that melanoma cells upregulate *T-RECS* in response to MEK-targeting therapy, and that *T-RECS* is a druggable vulnerability to either augment the anti-tumor response to MEK inhibition, or to restore therapeutic response in melanoma cells that have become resistant to MEK inhibition.

### *T-RECS* ASO treatment significantly suppresses melanoma tumor growth *in vivo*

**Figure 7:**
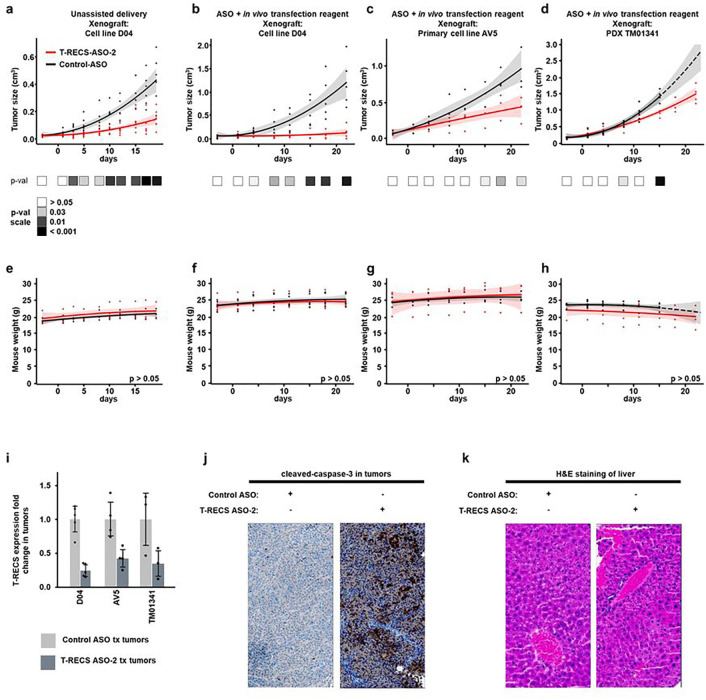
*T-RECS* ASO treatment significantly suppresses melanoma tumor growth *in vivo*. **a**) Significant tumor growth reduction was observed when comparing treatment groups for systemic (s.c.) treatment with either *T-RECS* ASO (red) or Control ASO (black) in mouse models carrying xenografts of the D04 melanoma cell line (3×200μg ASO/week, n=6). **b-d**) Significant tumor growth reduction was observed when comparing treatment groups for systemic (s.c.) treatment with either T-RECSASO (red) or Control ASO (black) in mouse models carrying xenografts of the b) D04 melanoma cell line (n = 5), **c**) AV5 primary melanoma cell line (n = 3) or **d**) TM01341 (n = 4) PDX patient derived tumor model (2×60μg ASO/week + *in vivo* transfection reagent jetPEI). **e-h**) The panels depict the progression of average mouse weight over time. Each panel corresponds to the weight trends of the treatment groups indicated above (**a-d**). No significant weight changes were observed between the *T-RECS* ASO and Control ASO groups at any time-point (p > 0.05). All growth and weight curves are presented as polynomial trend lines (Data for tumor sizes and weight over time are listed in **Suppl. Table 6**). i) *T-RECS* RNA levels were reduced in tumors of *T-RECS* ASO treatment groups compared Control ASO treatment groups at the end of study period of all cohorts shown in **b-d**). Tumours were harvested at end of treatment period and relative gene expression was normalized to Control ASO treated tumours. *T-RECS*RNA levels were reduced in D04 (0.25-fold, SD = 0.1), AV5 (0.44-fold) and PDX TM01341 tumours (0.34-fold). **j**) Immunohistochemical staining for the apoptosis marker Cleaved-Caspase-3 at the end of study period in tumors from Control ASO (left) or *T-RECS* ASO treatment (right) of treatment groups shown in panel **b**). **k**) Hematoxylin-eosin staining of mouse liver tissue after the end of the treatment period at the end of study period in tumors from Control ASO (left) or *T-RECS* ASO treatment (right) of treatment groups shown in panel **b**). Significance is shown as p-values calculated by Student’s t-test. * = p < 0.05, ** = p < 0.01, *** = p < 0.001.

To translate our findings *in vivo* and assess the potential clinical value of ASO intervention for melanoma, we tested the efficacy of *T-RECS* ASO treatment using melanoma cell line-derived xenograft and patient-derived xenograft (PDX) mouse models. We initially treated D04 tumor-bearing mice with 200μg subcutaneous ASO injection, three times a week (i.e., total: 600μg ASO/week), over the course of three weeks. We observed that the average tumor size was significantly smaller in the *T-RECS* ASO treatment group compared to the Control ASO group (Fig. 7a; p < 0.005).

Next, in order to reduce the dosage of ASO and follow the most recent advances in ASO-based therapy, we co-administered Control ASO or *T-RECS* ASO with an *in vivo* transfection reagent that is under clinical development (i.e., JetPEI^®^). We adjusted the treatment protocol down to 60μg subcutaneous ASO injection and twice a week (i.e., total: 120μg ASO/week), for three weeks. We tested this strategy using three tumor xenograft models, established from a melanoma cell line (D04), a primary melanoma cell line (AV5), and a NRAS^Q61^ melanoma PDX (TM01341). We found that this regimen induced significant tumor growth inhibition in tumors treated with *T-RECS* ASO in comparison to Control ASO across all models (Fig. 7b–d; p < 0.005, p < 0.04 and p < 0.003 in respectively D04, AV5, and TM01341).

Mouse weight, a surrogate to assess potential treatment toxicity, remained stable over the course of therapy for Control ASO and *T-RECS* ASO in all cohorts (Fig. 7e–h). We measured *T-RECS* lncRNA expression levels in tumors harvested from mice treated with *T-RECS* ASO versus Control ASO and co-administered with JetPEI, and we confirmed that *T-RECS* was significantly reduced in *T-RECS* ASO-treated D04, AV5 and TM01341 tumors (Fig. 7i). To test whether apoptosis was induced *in vivo* by *T-RECS*-targeting ASO treatment, we used immunohistochemistry to detect cleaved-caspase-3, a marker of apoptosis. We found that D04 xenograft tumors treated with *T-RECS* ASO systematically displayed greater levels of cleaved-caspase-3 staining in comparison to Control ASO treated tumors (Fig. 7j). Since ASOs have been reported to accumulate in the liver and cause toxic side effects, we also analyzed liver tissue for potential histopathologic changes by H&E staining and found no detectable hepatotoxicity across ASO-treated animals (Fig. 7k). Altogether, *these in vivo* results demonstrate that systemic treatment with an ASO that specifically targets the lncRNA *T-RECS*, is a well-tolerated and effective strategy to suppress the growth of MAPK pathway-mutated melanoma tumors.

## Discussion

Despite recent advancements in melanoma treatment, discovering therapeutically actionable mechanisms of melanoma progression remains essential to keep improving the outcome of patients.^2–4^ Here, we investigated whether particular long non-coding RNAs (lncRNAs) may be specifically associated with the tumorigenicity of NRAS-mutated/MAPK-driven melanoma, and whether such lncRNAs may be amenable for therapy using cutting-edge RNA-targeting interventions (ASO). Leveraging advanced genomic and proteomic methods, we found that MAPK-pathway activated melanomas display increased levels of expression of the lncRNA gene *AC004540.4*, which we named *T-RECS* for “Transcript REgulating Cell Survival”. We showed that *T-RECS* plays a pivotal role in repressing apoptosis of melanoma cells and tumors via regulation of pro-survival kinases and hnRNPA2/B1 functions. Our characterization of *T-RECS* as an actionable vulnerability shows that lncRNAs are therapeutically tractable, and that ASO-based RNA-targeting strategies can suppress the growth of NRAS/MAPK-driven melanoma tumors.

Therapies that target RNAs have opened a new clinical era. RNA targeting is a transformative approach because in principle it allows to target the product of any transcripted gene, whatever it may code for, thus immensely increasing the pool of druggable targets in any cell. The utility of ASO therapy is well-recognized to treat non-cancerous diseases and it is currently undergoing rigorous evaluation for cancer treatment in many clinical trials.^13,14^ Previous studies showed that ASOs can be more efficacious than siRNAs, especially to target nuclear RNAs.^19,35^ We found that the lncRNA *T-RECS* was significantly enriched in the nucleus of melanoma cells, and that targeting *T-RECS* with ASOs was highly effective to reduce the levels of *T-RECS* RNA and to inhibit melanoma cell growth and survival. *T-RECS* ASOs also performed systematically better than *T-RECS* siRNAs. Prior reports show that systemic ASO treatment can pose challenges, including limited ASO delivery and effectiveness^35^ as well as liver toxicity.^36^ To augment the binding affinity, stability, and target specificity, we modified the *T-RECS*-targeting GapmeR ASOs to incorporate LNAs and employed a fully modified phosphorothioate (PS) backbone.^37^ To reduce the risk of hepatotoxicity and increase delivery, we conducted *in vivo* efficacy experiments using unassisted and assisted delivery methods, the latter involving the transfection reagent JetPEI^®^. JetPEI is a reagent used in clinical trials for melanoma gene therapy that ensures robust, effective and reproducible RNA transfection (ClinicalTrials.gov ID: NCT04925713, NCT04853602, and NCT04160065). Both delivery methods significantly reduced melanoma tumor growth while causing no noticeable toxic side effects in mice, including in the liver. Importantly, co-administering JetPEI with *T-RECS* ASOs enabled us to greatly reduce the amount of ASO while effectively suppressing tumor growth (5-fold less ASO amount compared to unassisted delivery and at greater time intervals), thus further improving on the potential for treatment tolerability. In the perspective of additional clinical development, we envision that the versatility of ASOs to accommodate chemical modifications increasing drug bioavailability and pharmacokinetics ^35,37^, will allow to further enhance the anti-tumor effects of *T-RECS* ASOs while ensuring minimal toxicity.

To understand the mechanism of action of the T-RECS-targeting ASO treatment and how *T-RECS* may regulate the growth and survival of NRAS-mutated/MAPK-pathway driven melanoma tumor cells, we profiled the activity of kinase enzymes in melanoma samples. HT-KAM-derived kinome signatures revealed that treatment with *T-RECS*-targeting ASO specifically and significantly suppressed the activity of several kinases that promote cell survival and counteract apoptosis, such as AKT1, PIM1, CDK1, SRCs, PKA or PKC. This kinome reprogramming effect was not observed when treating melanomas with other ASOs that target other lncRNAs, including the known pro-oncogenic lncRNA MALAT1. Since these kinases play pivotal roles in orchestrating cell survival mechanisms,^26–30^ our results suggest that the lncRNA *T-RECS* may act as an central hub that regulates the intricate networks of pro-survival pathways, and confers a unique growth advantage to melanoma cells. This also suggests that, in order to blunt the complex signaling responses, that cancer cells engage and depend on to survive and keep proliferating, it may be therapeutically more effective to target *T-RECS* than any single one of these individual kinases or kinase-regulated pathways.

The current standard-of-care to treat melanoma heavily relies on drugs that inhibit the MEK and BRAF kinases to induce tumor regression by eliciting apoptotic cell death, or at minima to block tumor growth.^2–4^ These MAPK pathway-targeting therapies are often administered in combination regimens to reinforce the inhibition of the MAPK pathway, whether to induce a profound, upfront therapeutic response or to restore therapeutic sensitivity in relapsing melanomas.^4^ Our results suggest that such combinatorial targeted therapy strategy could be applied in the context of *T-RECS* dependency. Indeed, we found that treatment with *T-RECS* ASOs amplified the therapeutic efficacy of –and synergized with–the MEKi trametinib in NRAS-mutated melanoma tumor cells. As well, melanoma cells responded to MEKi treatment by increasing *T-RECS* expression, and MEKi-resistant melanoma cells remained sensitive to T-RECS ASO. Based on the kinome reprogramming triggered by *T-RECS* ASO and its significant impact on AKT1 and PIM1 activities, it is possible that jointly targeting MEK and *T-RECS* directly inhibits both the MAPK and AKT pathways, which are the two main drivers of therapeutic resistance and melanoma progression.^2,24^

Our study also revealed intricate regulatory connections between the lncRNA *T-RECS* and hnRNPA2/B1, a pro-oncogenic protein whose interaction networks includes nuclear-enriched lncRNAs.^38^ Treatment with *T-RECS* ASOs produced a rapid and striking reduction in hnRNPA2/B1 protein levels. We found a direct interaction between *T-RECS* and hnRNPA2/B1 proteins. In agreement with other studies that showed how lncRNAs can act as stabilizers of proteins,^39^ our findings suggest that *T-RECS* may stabilize hnRNPA2/B1 through their association in the nucleus. *T-RECS* and hnRNPA2/B1 exhibited elevated expression levels in melanoma, and their expression was significantly correlated in melanoma tissue specimens, while this was not observed in skin biopsies from healthy patients. Additionally, the upregulation of T-RECS coincided with elevated hnRNPA2/B1 protein levels, suggesting a potential reciprocal interaction between these molecules. Our findings suggest that the interactions between *T-RECS* and hnRNPA2/B1 may promote their pro-oncogenic effects in melanoma.

Most oncogenic lncRNAs, including *T-RECS*, exhibit strong tissue-specific expression patterns.^6^ While our study focuses on the role of *T-RECS* in NRAS-mutated/MAPK-driven melanoma cell survival, it likely has broader functions in other types of melanomas and cancers, as well as in pre- and non-malignant tissues.

Our data contribute novel insights into the previously uncharted territory of the lncRNA T-RECS. Our findings are the first to demonstrate an oncogenic function for T-RECS. Our results indicate that targeting *T-RECS* represents an untapped therapeutic opportunity to treat NRAS-mutated/MAPK-driven melanoma. Along with other recent studies, our work demonstrates that lncRNAs are promising targets for next-generation cancer therapy, and that ASOs can be used as emerging therapeutic tools to precisely target lncRNA molecules, opening a new window of therapeutic opportunities to treat melanoma.

## Materials And Methods

### Bioinformatic pipeline for identifying MAPK-responsive lncRNAs

#### Reference Annotation

A custom reference annotation of total 75,506 transcripts, referring to 35,101 genes, of which 16,405 were classified as non-coding, was built by integrating 13,870 lncRNA genes from the GENCODE^40^ (V19, July 2013 freeze, GRCh37, downloaded March 2015) into the RefSeq^41^ database (release 57, downloaded March 2013). Cuffcompare (version 2.1.1) was used to cut out redundant transcripts.

#### Assembly and identification of previously unidentified lncRNAs

After alignment to the human genome with TopHat (version 2.0.11), the reads were assembled into transcripts with Cufflinks (version 2.1.1). To discover novel lncRNAs, we excluded all transcript IDs that overlapped with any gene IDs from our initial reference annotation. To filter out transcriptional noise, we kept only multi-exonic transcript IDs which were > 200bp and had at least one intron region > 10bp. Next, isoforms were merged with Cuffcompare.

#### Coding Potential Assessment of Transcripts

To identify transcript IDs with a coding potential, we ran (a) the HMMER3 algorithm (considering all 6 open reading frames) to identify any protein family domain as noted in the Pfam database (release 27.0, Pfam-A and Pfam-B domains considered) and (b) the Coding Potential Assessment Tool (CPAT v1.2.1).

#### Filter for DE genes

Cuffdiff (v.2.1.1) was used to identify differential gene expression analysis between PHM^E^ and PHM^Q61^. From a reference of 35,905 genes, we discarded genes with FPKM < 0.2 in both conditions and kept genes with log2-fold change > 1 or < −1. Cufflinks was used to obtain FPKM-values in RNA-Seq Data from the D04 and MM415 melanoma cell-lines. Log2 transformations were performed to calculate expression fold change in the comparisons: PHM^E^ vs. PHM^Q61^, PHM vs. D04, and PHM vs. MM415. Genes that had a log 2-fold change > 1 or < −1 and showed same tendency in all three comparisons were considered as differentially expressed.

#### TCGA data extraction and processing

Raw .*fastq* files were obtained from The Cancer Genome Atlas (TCGA) from 86 NRAS mutant melanoma patients from the TCGA-SKCM dataset. Transcript de-novo assembly was performed as described above.

### Viral transduction

NRAS^Q61R^ cDNA was cloned into the Gateway entry vector pENTR/D-topo. pENTR/D-topo-NRAS^Q61R^ was subjected to site-directed mutagenesis to generate mutants which were then validated by Sanger-sequencing. NRAS^Q61R^ cDNA in pENTR was cloned into the Gateway cloning-enabled destination vector gFG12. After lentiviral transduction, cells were grown for two weeks followed by cell sorting facilitated by GFP expression intensity on a FACS Aria II cell sorter.

### Sanger-Sequencing

RNA from PHM cell-lines was extracted using Purelink^™^ RNA extraction kit (Thermo Fisher Scientific^®^) and transcribed into cDNA. Sanger-Sequencing was performed by Quintarabio Inc. For NRAS amplification, the forward primer CGCACTGACAATCCAGCTAA and the reverse primer TCGCCTGTCCTCATGTATTG were used.

### Expression analysis in TCGA and GTEx

The comparative analysis of TCGA and GTEx gene expression data was done in R. For TCGA data, the SKCM dataset (n = 469) was used and filtered for patients with either NRAS- or BRAF-mutated melanoma (n=366). The GDCquery function of the TCGAbiolinks package was run with the following parameters: project =“TCGA-SKCM”, data.category =“Transcriptome Profiling”, data.type =“Gene Expression Quantification”, workflow.type =“HTSeq – FPKM”. GDCdownload and GDCprepare then produce a RangedSummarizedExperiment. Expression values are then stored in a data frame and converted to TPM by dividing each FPKM-value by the total FPKM of each sample and multiplying by 10^6. To retrieve GTEx data, “GTEx_gene_tpm.gct” was downloaded from gtexportal.org/home/datasets.^42^ This was then filtered for skin samples by retrieving the GTEx IDs for skin samples from “GTEx_v8_Skin.csv” from the same website (n=1305). Removing the version numbers from the gene names converted them to their corresponding Ensembl gene IDs.

The raw read counts were converted to TPM-values. For both TCGA and GTEx, duplicate genes were removed. If a patient provided multiple specimens, only the first would be used. The ensemble ID for *T-RECS* were ENSG00000225792 and ENSG00000122566 for hnRNP2/B1. Cor.test was applied to find the correlation between each gene and *T-RECS*, and the same for hnRNPA2/B1. Spearman's correlation coefficient (ρ) was used to measure rank correlation. 2000 random genes were sampled from both TCGA and GTEx datasets. The correlation of *T-RECS* and hnRNPA2/B1 was ranked against the correlations with 2000 random genes, divided in 10 groups of 200 genes.

### Cell culture

Human melanoma cell-line VMM39 was purchased from American Type Culture Collection (ATCC^®^). Human melanoma cell-lines D04, MM415, WM1366, WM3629, WM3211, Sk-Mel-2 and Sk-Mel-28 were gifted by Dr. Boris Bastian at the UCSF. Primary human melanoma cell-line Hs852.T, was purchased from the Cell and Genome Engineering Core (CGEC) at the UCSF. Primary human melanoma cell-line AV5 was obtained from metastasis of a BRAF-mutated melanoma patient. Detailed information for cell-lines is listed in **Suppl. Table 1**. Cell culture related research was approved by UCSF Human Research Protection Program Institutional Review Board (IRB# 12–0948) and carried out in accordance with relevant guidelines and regulations. The Resistant cell-line D04RM was established as previously described.^8^ Primary human melanocytic cell-lines (PHM) from infant foreskin of healthy donors were available in the Ortiz’ lab cell repository. Melanoma cell-lines were maintained in RPMI 1640 media supplemented with 10% (vol/vol) heat inactivated fetal bovine serum. Melanocytes were maintained in M254 medium with HMGS supplements (1x final solution). All cell-lines were incubated at 37 °C under 5% CO2.

### Antisense Oligonucleotide synthesis and transfection

Primer sequences TCACTATAGGGAGAGACACTCAAAGCCTGAGTAACAGA and TCACTATAGGGAGACTGACTGAGATTTTATTGAGCTGTG were used to create *T-RECS* targeting esiRNA following a standard protocol.^43^. *AC004540.4* SiRNA-1 (guide strand sequence: ACAAAGAGAGACAGGAAAUUU) was purchased from Horizon Discovery Biosciences Ltd and designed using the siDESIGN software. *AC004540.4* siRNA-2 (guide strand sequence: UAACUAUUAGCUUCAUGUUUUUACCCA) and *AC004540.4* siRNA-3 (guide strand sequence: AUCACUGAAUUGACAUGCUGUUGGCAG) were designed by and purchased from Integrated DNA Technologies, Inc (IDT). For pooled non-targeting Control siRNA design, the guide strand sequences UGGUUUACAUGUCGACUAA, UGGUUUACAUGUUGUGUGA, UGGUUUACAUGUUUUCUGA and UGGUUUACAUGUUUUCCUA were used. The *AC004540.4* ASO-1 (sequence: GACTGGAGATAATTAA), *AC004540.4* ASO-2 (sequence: TGCGCGGCGGAAAGAA), MALAT1 ASO (sequence: TAAAGCCTAGTTAACG) and hnRNPA2/B1 ASO (sequence: GACCGTAGTTAGAGG) GapmeRs were purchased from QIAGEN N.V. and designed using the GeneGlobe design and analysis hub. The *AC004540.4* ASO-3 (sequence: CTCATGAGCTGTCGTA) GapmeR was designed by and purchased from IDT. For non-targeting Control ASO design, the QIAGEN N.V. standard sequence AACACGTCTATACGC was used. In all experimental procedures, a consistent antisense oligonucleotide concentration of 50nM was used, unless specified otherwise. The transfection reagent Lipofectamine^™^ 3000 (2ul/ml) was added according to the manufacturer’s instructions.

### Cell growth analysis

Dependent on cell doubling time, 0.7–2 × 10^^3^ cells were seeded in 96 well-plates one day prior to treatment. Cells were treated with ASOs for five days unless specified otherwise. Total luminescence was measured on the Synergy^™^ HT (Agilent Technologies Inc) plate reader using Promega^®^ CellTiter-Glo^®^ and Gen5 software. Cell growth was normalized to Control ASO treatment.

### Colony formation Assay

Dependent on cell doubling time, 1–2 × 10^^3^ cells were seeded in six well-plates one day prior to treatment. Six days after transfection, cells were washed with PBS, fixed with 10% neutral buffered formalin, and stained with 0.1% crystal violet solution. Colonies were defined as cell conglomerates with > 50 cells. Digital images of plates were evaluated by two independent reviewers for colony counts. The final counts were calculated as the average count of both reviewers for all triplicates.

### RNA extraction and quantitative real-time PCR (qRT-PCR)

TRIzol^™^ Solution (Thermo Fisher Scientific^®^), Phenol:chloroform:isoamyl alcohol (MilliporeSigma^®^) or NucleoSpin^®^ RNA kit (Takara Bio USA, Inc.) were used for extracting Total RNA from cells and tissues according to the manufacturer’s instructions. Total RNA was quantified by NanoDrop^™^ ND-1000 (Thermo Fisher Scientific^®^) or Quibit^™^ 4 (Thermo Fisher Scientific^®^). 50ng or RNA was reverse transcribed using the cDNA synthesis and gDNA removal QuantiTect^®^ Reverse Transcription Kit (Thermo Fisher Scientific^®^). Real time PCR was performed using the iTaq^™^ Universal SYBR^®^ Green Supermix (Bio-Rad Laboratories, Inc.), 20ng of cDNA and on a QuantStudio^™^ 5 Real-Time PCR System or a 7500 fast real time PCR system (both from Thermo Fisher Scientific^®^). Relative gene expression was calculated using the comparative Ct method, normalized to GAPDH or β-actin. Primers are listed in **Suppl. Table 7**. Primers were obtained from IDT.

### In situ hybridization and Immunofluorescence

In situ hybridization analyses were conducted using the RNAscope Multiplex Fluorescent Reagent Kit version 2 system, using custom-made probes to *AC004540.4* (*T-RECS*). Following RNAscope, sections were immediately processed for immunofluorescent staining with hnRNPA2/B1. The Multiplex Fluorescent Reagent Kit version 2 was used according to the manufacturer's instructions. Immunofluorescent staining was performed using primary antibody dilutions of 1:200 for hnRNPA2/B1 (ProteinTech 14813–1-AP), incubating overnight at room temperature. Sections were treated with secondary antibodies of donkey anti-rabbit AlexaFluor-Plus 555 (1:400; Invitrogen) and DAPI for 2 hours prior to imaging. TSA Cy5 fluorophores were used to amplify the fluorescent signals for *T-RECS*, in accordance with the instructions in the Multiplex Fluorescent Reagent Kit version 2. 100nM final ASO concentration for 1 day was used for cells that received treatment.

### Fluorescence Imaging

Fluorescence imaging was performed using a Zeiss Axio Observer Z1 with a 20X objective. All images for assessment of each outcome measure were captured at a constant exposure, using identical microscope settings.

### Purification of Nuclear and Cytoplasmic RNA

Total nuclear and cytoplasmic extracts were obtained using the SurePrep^™^ Nuclear/Cytoplasmic RNA purification kit (Thermo Fisher Scientific^®^) according to the manufacturer’s instructions. RNA extraction and qRT-PCR were performed as described above.

### RNA-sequencing

Total RNA was isolated using the RNeasy^®^ mini-Kit (QIAGEN N.V.) following the manufacturer’s protocol. Quality check for extracted RNA was done using 2100 Bioanalyzer (Agilent Technologies Inc.) or Tapestation 4200 System (Agilent Technologies Inc.). All samples had a RIN score > 8. For samples used for identification of MAPK-responsive lncRNAs, cDNA-sequencing libraries were prepared using the Illumina^®^ TruSeq^®^ Total RNA Sample kit and paired-end, 101-bp sequencing was performed by Centrillion Genomic Services (Centrillion Tech.) on an Illumina^®^ HiSeq^®^ 2000. Sequence reads were aligned to the human genome (hg19) using TopHat (Version 2.0.11). For DE gene analysis of ASO-transfected D04 samples (3 days of either Control ASO or *AC004540.4* ASO-1 incubation), NEBNext^®^ ultra-RNA library prep kit (New England Biolabs^®^ Inc.) with rRNA depletion and paired-end, 2×150-bp sequencing was performed by Genewiz^®^ on an Illumina^®^ HiSeq^®^ 4000. Sequence reads were aligned to the GRCh38 reference genome.

### Analysis of *T-RECS* ASO treatment induced DE genes

Differential expression (DE) analysis was done using DESeq2. Differentially expressed genes were defined by more than 1.5-fold changes (log_2_ > 0.58 or < −0.58) in expression with FDR < 0.05. Pathway enrichment analysis was done using DAVID Functional Annotation Clustering analysis (version 6.8).^44,45^

### Kinase activity mapping technology

For samples to be analyzed with the HT-KAM platform, cells were treated with ASOs for 24 hours and at ~85% confluency cells were washed three times with cold PBS and lysed with freshly prepared 1X cell lysis buffer (1ml per 2.5×10^6^ cells) (10x Cell lysis buffer, Cell Signaling Technology^®^, Cat.No. 9803), complemented with 1x Halt Protease and Phosphatase (Thermo Fisher Scientific^®^ Cat.No 1861281). Lysates were scraped off, spun down at 14,000 rpm. at 4°C for 15 min and supernatants were stored at −80°C. The high throughput kinase activity mapping (HT-KAM) platform uses arrays of peptides that act as sensors of phosphorylation activity.^24^ The phospho-catalytic signature of samples is established from simultaneously occurring ATP-consumption tests measured in the presence of individual peptides that are experimentally isolated from each other. Assays were run in 384 well-plates, where each experimental well contains one peptide. The final 8μL reaction mixtures per well contain: (a) kinase assay buffer (1X KaB: 2.5mM Tris-HCl (pH7.5), 1mM MgCl_2_, 0.01mM Na_3_VO_4_, 0.5mM - glycerophosphate, 0.2mM dithiothreitol (DTT), prepared daily; (10X KaB Cell Signaling Technology^®^, Cat.No. 9802), (b) 250nM ATP (prepared daily with 1X KaB; Cell Signaling Technology^®^ Cat.No. 9804), (c) 200μg/ml 11-mer peptide (lyophilized stocks originally prepared as 1mg/ml in 1X KaB, 5% DMSO), and (d) samples made from cell at ~10μg/ml total protein extract. Samples are kept on ice and diluted in 1X KaB < 30min before being used. Controls with no-ATP, or no-peptide, or no-sample as well as ATP standards are run side-by-side within each 384 well-plate. High-throughput liquid dispensing of all reagents is performed using a Biomek^®^ FX Laboratory Automation Workstation from Beckman Coulter. All reagents are kept on ice and plates on cold blocks until enzymatic reactions are started. Once the dispensing of the reaction mixtures is complete, the plates are incubated for 1h at 30°C. ATP is detected using Kinase-Glo revealing reagent (Promega^®^; Cat.No V3772), which stops the activity of the kinases and produces a luminescent signal that directly correlates with the amount of remaining ATP in the samples. Luminescence is acquired using the Synergy 2 Multi-Mode Microplate Reader from BioTek. Luminescence data are inversely correlated with the amount of kinase activity. For a more detailed description of the peptide sensors design, sequence and connectivity between peptides and kinases, as well as data normalization steps and analysis, refer to: ^22,24,25^. The activity of kinase enzymes is derived from their respective subset of biological peptide targets included in the assay.

### Caspase Glo 3 & 7 assay

Dependent on cell doubling time, 2–3 × 10^^3^ cells were seeded in 96 well-plates one day prior to transfection. One day after transfection Total luminescence was measured on the the Synergy^™^ HT (Agilent Technologies Inc) plate reader using The Promega^®^ Caspase-Glo^®^ 3/7 Assay and Gen5 software. Experiments were performed in quadruplicates.

### Annexin V assay

1 × 10^^5^ D04 cells were seeded in six well-plates one day prior to transfection. One day after transfection live, dead, and apoptotic cells were differentiated using the Invitrogen^™^ Dead Cell Apoptosis Kits with Annexin V (Cat. no: V13241), following manufacturers protocol. Cells were sorted using a BD^®^ LSR II Flow Cytometer.

### Protein extraction and immunoblotting

1 × 10^^5^ D04 cells were seeded in six well-plates one day prior to transfection. Total protein lysates were homogenized in 1x RIPA buffer and Halt protease and phosphatase inhibitor cocktail (Thermo Fisher Scientific^®^) followed by centrifugation at 14,000 RPM/minute at 4°C. Protein concentration was quantified using the Pierce^™^ BCA Assay Kit (ThermoFisher Scientific^®^). Linear absorbance was measured using the Synergy^™^ HT (Agilent Technologies Inc) plate reader. Total protein in 1× Laemmli buffer with 10% 2-mercaptoethanol was separated by SDS/PAGE, transferred for 15 h to a PVDF membrane (IPVH00010; MilliporeSigma^®^) by electroblotting with 20% (vol/vol) methanol, and blocked for 1 h in in Intercept (TBS) blocking buffer (LI-COR^®^). Membranes were incubated overnight at 4 °C with primary antiserum for hnRNPA2/B1 (Abcam^®^, cat.no.: ab31645, dilution 1:750) and b-Actin (Cell Signaling Technology^®^, cat.no.: 8457, dilution 1:2500) following incubation with secondary Goat Anti-Rabbit serum (LI-COR^®^, dilution 1:5000) for 1 h and scanned using the Li-COR^®^ Odyssey^®^ Imaging system. Protein expression was quantified using Image Studio Lite Version 5.2.5

### RNA-Binding Protein Immunoprecipitation

The Magna RIP^™^ Kit (MilliporeSigma^®^) was used following standard protocol. 10μg of Antibody for Rabbit IgG (MilliporeSigma^®^, Cat.no.: PP64B) and hnRNPA2/B1 (Proteintech^®^, Cat.no.: 14813–1-AP) were used to load magnetic beads. RNA precipitate was subjected to qRT–qPCR analysis.

#### Calculation of Combinational Index (CI)

The effects of drug combinations on cell growth were assessed by calculating the combination index (CI) = log2[*E*_a,b_/(−*E*_a_x*E*_b_)], where *E*_a_ and *E*_b_ correspond to the effects of drugs A and B alone at a given concentration, and *E* a,b corresponds to the combined effects of drugs A and B at the same concentration, and a combination index of < 0 indicates synergy while a combination index of > 0 relates to an antagonistic effect. The individual combination indices per drug combination were then averaged. Cells were treated for three days.

### Animal models

Rodent experimental procedures were approved by the Office of Research institutional Animal Care and Use Program (IACUC, Chair: Jeremy Lieberman, MD) at the University of San Francisco (UCSF). All *in vivo* studies were conducted under the authorized protocol number AN174613–03. Mice were maintained in a pathogen free environment and had free access to food and water. For PDX tumor models, the PDX type TM01341, derived from liver metastasis of a male melanoma patient, was engrafted on 4- to 6-week-old NOD.Cg-Prkdc^scid^ Il2rg^tm1Wjl^/SzJ mice (Stock.no 005557) on the right posterior dorsal flank (n = 4/group). For cell-line models, 2×10^^6^ D04 (n = 5/group) and AV5 (n = 3/group) cells in 150μl of PBS and 50μl of Matrigel were subcutaneously injected on the posterior dorsal flanks of 4- to 6-week-old homozygous nude Foxn1^nu^/Foxn1^nu^ mice (Stock.no 007850). Mice and PDX tissue were obtained from JAX^®^. Tumor size was measured using a digital caliper and the formula 0.5 × (length × (widtĥ2)) was used to calculate tumor volume. For unassisted delivery, mice were treated 3x/week with 200μg of ASOs diluted in an overall amount of 100μl PBS. For assisted delivery, mice were treated twice a week with 60μg of ASOs and 9.6μl of *in vivo* JetPEI^®^ diluted in an overall amount of 200μl 5% glucose. ASO injections were applied subcutaneously. Mice were weighted at the days of treatment and observed for signs of distress or disorder. Mice were euthanized after three weeks of ASO application or when tumors reached a diameter of > 2cm. All experiments were performed in accordance with the UCSF Laboratory Animal Resource Center (LARC) guidelines. After euthanasia, tumor samples and liver tissue were excised and fixed in formalin solution, followed by storing in 70% ethanol and Immunohistochemistry staining. Tumor samples were also placed in RNAlater^™^ Stabilization Solution (Thermo Fisher Scientific^®^) and stored at −20°C. Invitrogen^™^ TRIzol^™^ Solution (Thermo Fisher Scientific^®^) was used to extract RNA from tissue and qRT-PCR was performed to analyze gene expression.

### Immmunohistochemistry

Tumor tissues were extracted from mice immediately after euthanasia and fixed in 10% neutral buffered formalin for 24 hours, followed by storage in 70% EtOH. Histopathology was conducted by the UCSF Histology and Biomarker Core. FFPE sections were collected at 4-micron thickness and mounted on center of positively charged glass slides (Superfrost Plus Slides Fisher Item# 12–550-15) following lab standard procedures for the sectioning of paraffin blocks. Slides where air-dried overnight for 8 to 24 hours then baked and deparaffinized. For hematoxylin and eosin (H&E) staining, slides were baked at 60°C for 15 minutes prior to staining; for immunohistochemistry (IHC) staining, slides were baked overnight at 60°C prior to staining. H&E staining was performed on the Leica XL Autostainer using hematoxylin (part no. 6765015 from Thermo Scientific) for seven minutes and alcoholic eosin (Part No. 6765040 from Thermo Scientific) for 20 seconds. IHC was done on the Roche Ventana Discovery Ultra Autostainer using antibodies for Cleaved-Caspase-3 (Cell Signaling Technology^®^, cat.no.: 9664S, dilution 1:600). Ventana Protocol Summary: CC1 cell conditioning at 95°C for 24 minutes, Inhibitor time 12 minutes, primary antibody incubation at 36°C for 60 minutes, anti-Rabbit HQ detection at 37°C for 12 minutes, Chromagen: DAB. The Zeiss Axio scanner was used for digital slide scanning with brightfield images collected at 20X magnification using Zeiss Zen software.

### Statistics and reproducibility

Error bars in all the plots indicate mean ± S.D. A p-values were calculated by Student’s t-test and were considered significant when < 0.05. All experiments were performed at least three times, unless otherwise indicated. Statistical tests were calculated with Microsoft^®^ Excel Version 2107.

### Tools for figure generation

Bar plots and volcano plots were created with Python version 3.10 using the Matplotlib (v3.8) and Seaborn (v0.12) libraries.^46,47^

## Data Availability

RNASeq data presented in this study is deposited in the Gene Expression Omnibus (Accession ID: GES197623). Patient derived data was accessed from the public domain resources https://www.cancer.gov/tcga and https://gtexportal.org.
